# Acute myeloma kidney and SARS-COV2 infection with dialysis need: never say never - a case report

**DOI:** 10.1186/s12882-023-03237-8

**Published:** 2023-07-06

**Authors:** Gabriele Donati, Agnieszka Przygocka, Fulvia Zappulo, Gisella Vischini, Sabrina Valente, Gaetano La Manna

**Affiliations:** 1 Nephrology Dialysis and Renal Transplantation Unit, Azienda Ospedaliero-Universitaria di Modena. Surgical, Medical, Dental and Morphological Sciences Department (CHIMOMO), University of Modena and Reggio Emilia, Modena, Italy; 2 Nephrology Dialysis and Renal Transplantation Unit, Department of Experimental Diagnostic and Specialty Medicine (DIMES), University of Bologna, IRCCS Azienda Ospedaliero Universitaria di Bologna, Via Massarenti 9, 40138 Bologna, Italy; 3 Clinical Pathology, Department of Experimental, Diagnostic and Speciality Medicine (DIMES), University of Bologna, Bologna, Italy

**Keywords:** AKI, Sars-CoV-2, Multiple myeloma, Dialysis, Case report

## Abstract

**Background:**

Older individuals with multiple comorbidities and especially patients with multiple myeloma are at higher risk of contracting SARS-CoV-2. When patients with multiple myeloma (MM) are also affected by SARS-CoV-2 the time to start immunosuppressants is still a clinical dilemma especially when urgent hemodialysis is required for acute kidney injury (AKI).

**Case presentation:**

We present a case of an 80-year-old woman who was diagnosed with AKI in MM. The patient began hemodiafiltration (HDF) with free light chain removal combined with bortezomib and dexamethasone. The reduction of free light chains concurrently was obtained by means of HDF using poly ester polymer alloy (PEPA) high-flux filter: 2 PEPA filters were used in series during each 4-h length HDF session. A total of 11 sessions was carried out. The hospitalization was complicated with acute respiratory failure caused by SARS-CoV-2 pneumonia successfully treated with both pharmacotherapy and respiratory support. Once the respiratory status stabilized MM treatment was resumed.

The patient was discharged in stable condition after 3 months of hospitalization. The follow up showed significant improvement of the residual renal function which allowed interruption of hemodialysis (HD).

**Conclusions:**

The complexity of patients affected by MM, AKI, and SARS-CoV-2 should not discourage the attending physicians to offer the adequate treatment. The cooperation of different specialists can lead to a positive outcome in those complicated cases.

## Background

Older individuals and patients with comorbidities such as hypertension, heart disease, diabetes, lung disease, chronic kidney disease (CKD), and cancer are at higher risk of contracting this severe infection and deteriorating of general conditions. We report the case of AKI secondary to MM and severe COVID-19 infection.

## Case presentation

An 80-year-old woman was admitted in our ward because of AKI. Her past medical history was remarkable for type 2 diabetes mellitus in therapy with metformin; hypertension; breast cancer treated with mastectomy currently in remission; deep vein thrombosis diagnosed in 2014 in therapy with apixaban. She reported decrease in urine output and dyspepsia. A physical examination showed mild pedal oedema and bibasilar crackles on lung auscultation. Hemodynamic parameters were within the normal values. On admission laboratory analysis revealed the following values: serum creatinine level, 9 mg/dL (estimated glomerular filtration rate [eGFR] of 4 ml/min); blood urea nitrogen, 232 mg/dL; haemoglobin, 9.1 g/dL; leukocyte count, 5060/mm^3^; platelet count, 118,000/mm^3^; erythrocyte sedimentation rate, 48 mm/h; C-reactive protein (CRP), 1.12 mg/dL; albumin, 28.9 g/L; potassium, 4.2 mmol/L; albumin-adjusted calcium, 10.8 mg/dL. Blood gas analysis was consistent with lactic acidosis. Urgent HD treatment was performed through central venous catheter.

Further laboratory tests were remarkable for 24-h proteinuria of 1,700 mg /day. Serum protein electrophoresis was positive for a monoclonal spike in the beta region. Urine protein electrophoresis detected kappa light chains. Serum free light chains (sFLC) were kappa light chains of 2894.7 mg/L and lambda light chains of 16.4 mg/L with kappa: lambda ratio of 176.5.

**Fig. 1:**
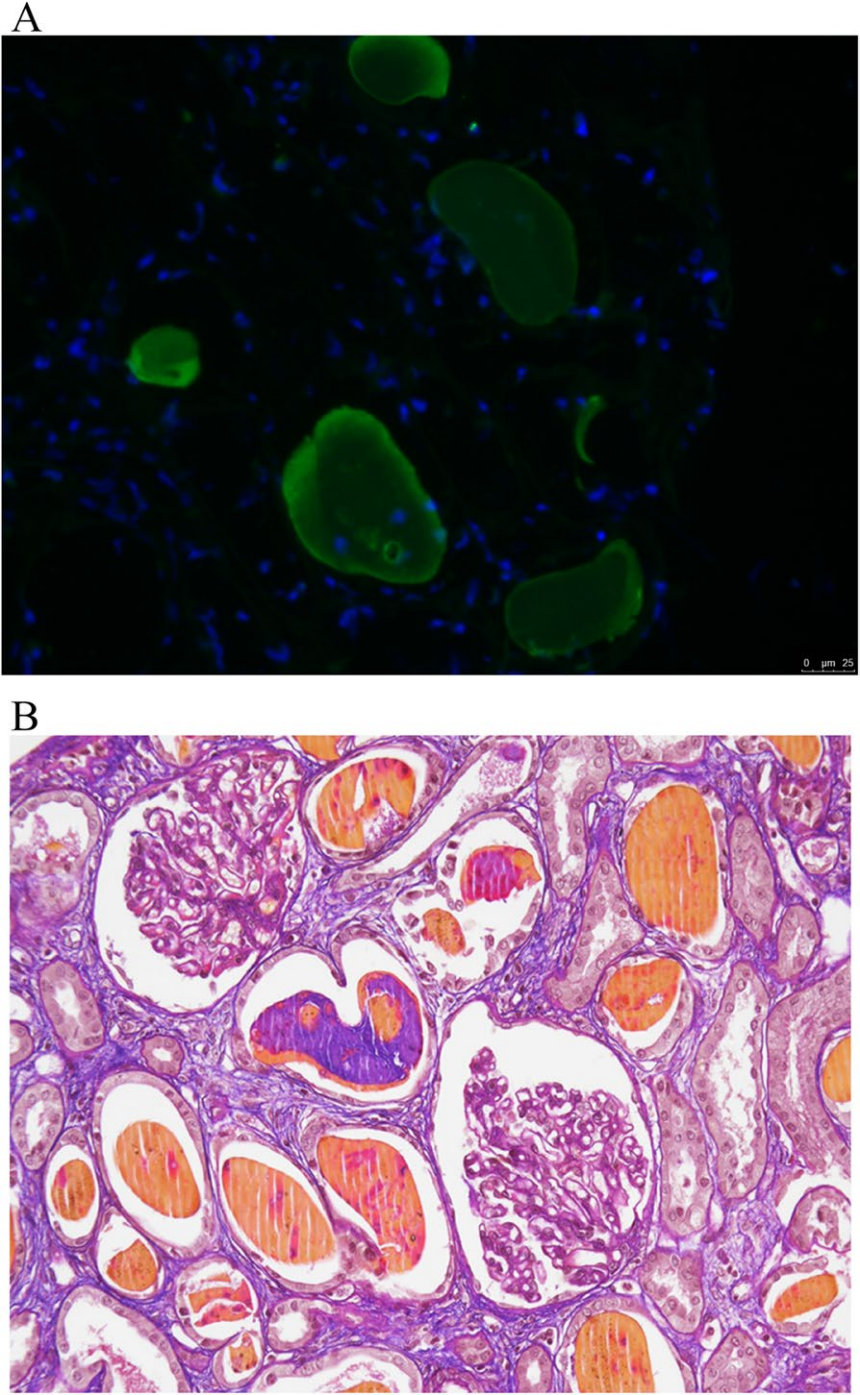
**A** Pronase digestion of the paraffin tissue by denaturized cell membranes demonstrated the presence of intracytoplasmic monoclonal kappa light chains in the tubular cells. **B** Acid Fucsin Orange G (AFOG) staining (4x) demonstrated the presence of intratubular casts helping the diagnosis of Myeloma Cast Nephropathy

**Fig. 2:**
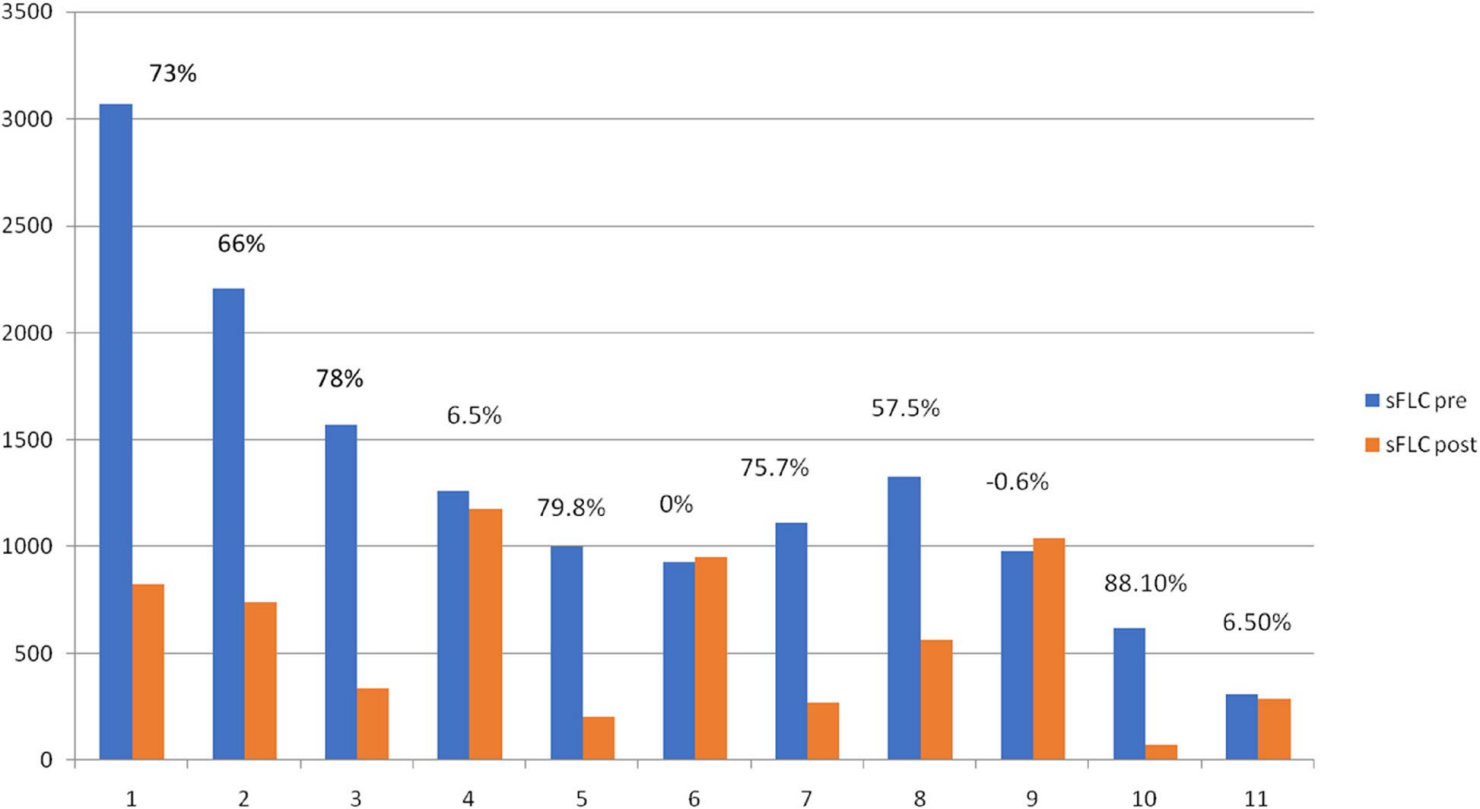
Serum level of K-FLC Before and after each dialysis session. Percentages represented in the figure are referred to the reduction ratio per session (RR) for K-FLC, RR = (T0—T1-corr)/ T0 × 100. T0 was K FLC level before dialysis, T1 at dialysis end. The values measured at T1 were corrected for haemoconcentration due to the patient’s weight loss according to Bergström and Wehle’s formula [2]

On day 8 renal biopsy was carried out (delay in execution of the biopsy was caused by initial alteration of coagulation parameters after immediate discontinuation of apixaban): the immunofluorescence staining showed polyclonal free light chain deposition in the tubular cells. Congo red staining did not detect the presence of amyloid. In the light of strong suspicion of cast nephropathy, the immunofluorescence analysis with the use of pronase enzyme was performed. Pronase digestion of the paraffin tissue by denaturized cell membranes demonstrated the presence of intracytoplasmic monoclonal kappa light chains in the tubular cells (Fig. 1A). It allowed to confirm the diagnosis of cast nephropathy (CN) which is the most common renal complication of MM (Fig. 1B). Positron emission tomography (PET) did not show extramedullary sites of MM. Given the age of the patient, COVID-19 pandemic emergency as well as laboratory and renal biopsy results haematologist concluded that bone marrow biopsy was not necessary to confirm the diagnosis of MM. The patient started therapy with bortezomib and dexamethasone on day 10. To improve the reduction of free light chains concurrently she received extracorporeal treatment with hemodiafiltration (HDF) using poly ester polymer alloy (PEPA) high-flux filter (Nikkiso FDY 210 GW, surface area 2.1 m^2^, cut off 32,600 daltons) (Fig. 2). To enhance sFLC removal by adsorption, two filters were used in series during each 4-h length HDF session. Vascular access was a temporary central jugular venous catheter (16 cm long, diameter 11.5 French, Mahurkar®, Medtronic, Minneapolis, USA). The patient received 11 extracorporeal treatments by means of online HDF. All treatments were well tolerated. Mean blood flow was 260 ± 20 ml/min, while median convective volume was 18 (15–20) L/session. The mean ultrafiltration rate was 330 ± 30 ml/h. Low molecular weight heparin 4000 IU (enoxaparin sodium Inhixa™, Techdow Pharma, Milan, Italy) was started as anticoagulation during dialysis and as prophylaxis of deep venous thrombosis every other day. Enoxaparin was administered in a single bolus on starting dialysis in the arterial line before the filter [1]. No clotting of the circuit was observed during all the HDF sessions carried out, also owing to the filter change at the second hour.

Bortezomib was administered the day after the HD session (Velcade® 1.3 mg/ m^2^, Janssen Cilag Spa, Beerse, Belgium). Those interventions resulted in improvement of urine output until 2.5 L/day.

**Fig. 3:**
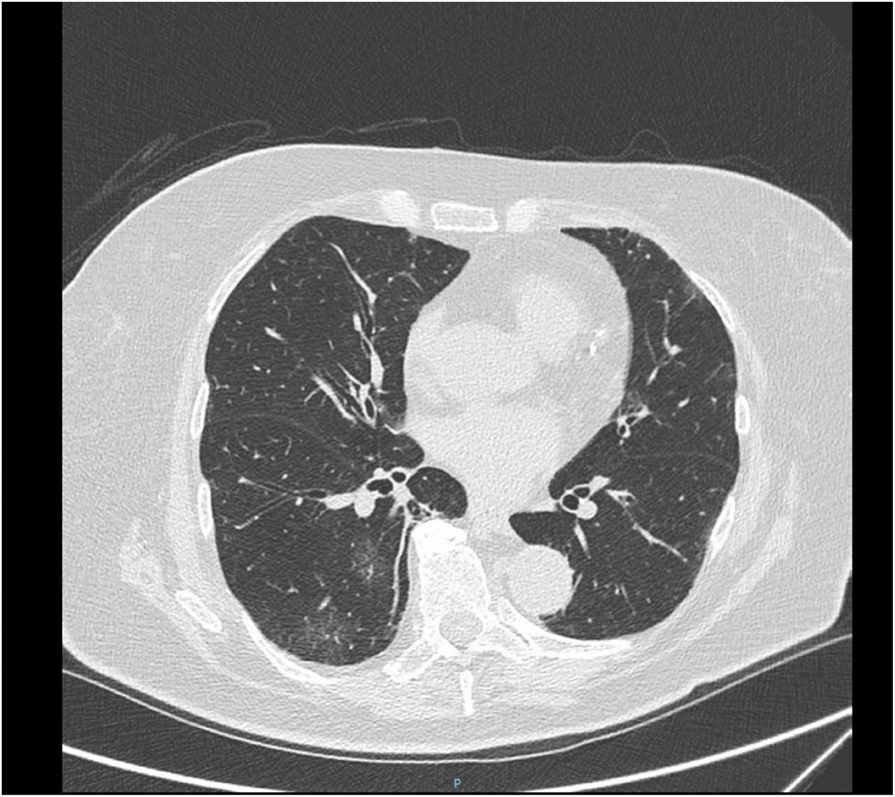
The first HRCT which shows mild pneumonia signs

On day 19 (9 days after starting chemotherapy) the patient contracted SARS-CoV-2 infection diagnosed through nasopharyngeal swabs. The first High-resolution computed tomography (HRCT) showed initial interstitial pneumonia (Fig. 3). The respiratory rate was 22 breaths per minute and oxygen saturation was 99%. Arterial blood gas analysis (ABG) revealed a pO2 of 76 mmHg, pCO2 of 38 mmHg and P/F (pO2/FiO2 ratio) of 361. No other symptoms were detectable. Laboratory studies were notable for WBC 4090/mm^3^ with lymphopenia of 540/mm^3^.

She started therapy with hydroxychloroquine and antimyeloma treatment was stopped but high-dose intravenous steroid was administered.

**Fig. 4:**
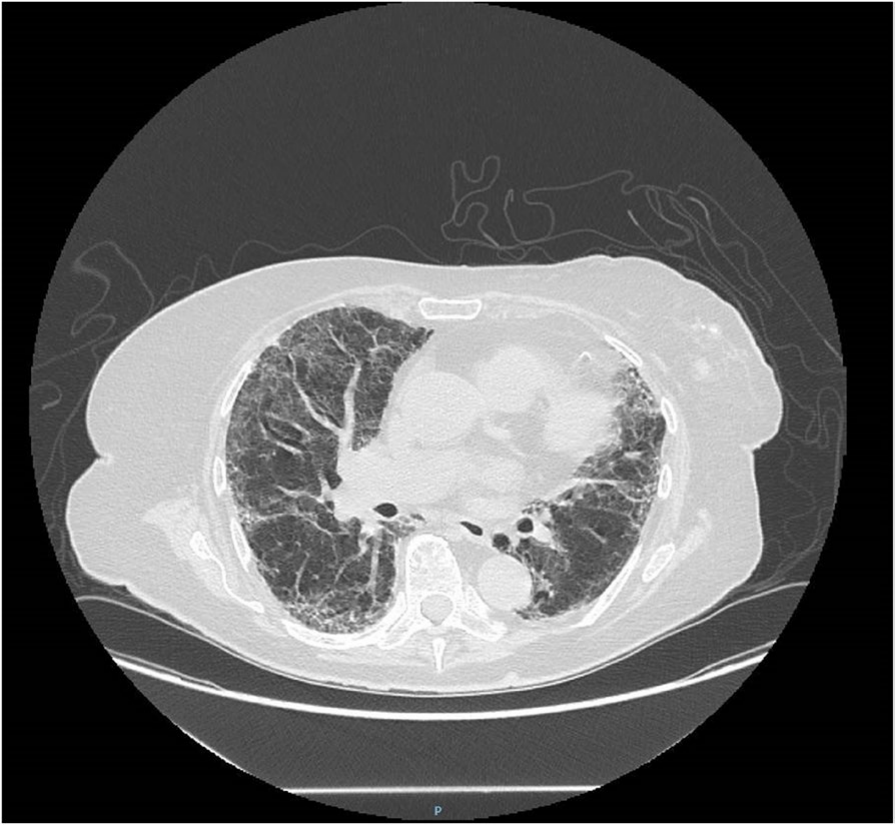
The second HRCT with severe and bilateral Sars Cov2 interstitial pneumonia

On day 26, after the diagnosis of SARS-CoV-2 infection, the patient presented acute dyspnoea. ABG revealed hypoxemia. HRCT was repeated and demonstrated severe worsening of the interstitial pneumonia with increase of ground glass areas in both lungs in central as well as peripheral regions (Fig. 4). Due to deterioration of respiratory condition the patient was treated with CPAP (Continuous Positive Airway Pressure) and high dose steroid in the Nephrology Unit, as because of her comorbidities she was not admittable in the Intensive Care Unit. She continued CPAP treatment for 3 weeks (PEEP 10 cm H_2_O 16 h per day) with progressive improvement of the oxygenation and respiratory status. Therefore, the patient required to continue HDF treatment for AKI, aside from the primary goal of sFLC removal. Once the respiratory status stabilized, after haematological re-evaluation, despite the persistence of SARS CoV-2 positivity MM treatment with bortezomib was resumed from day 44 in consideration of risk/benefit ratio. In the light of resolution of all symptoms and testing with nasopharyngeal swabs resulting negative the patient was considered recovered from SARS-CoV-2 infection only on day 105 of hospitalization.

The patient continued therapy with bortezomib and dexamethasone (in total during the hospitalization she received 3 cycles of therapy) and extracorporeal light chains removal treatment with optimal reduction of sFLC < 500 mg/L (kappa FLC serum level at the time of discharge: 93 mg/L).

The patient was discharged in good condition after 3 months of hospitalization. Subsequently she recovered renal effective urine output and renal function to be no longer dependent on dialysis treatment. Five months afterwards the laboratory studies showed further improvement with serum creatinine of 1.6 mg/dL, blood urea nitrogen of 84 mg/dL and proteinuria within the physiological limits (240 mg/day). The patient continues haematological therapy with bortezomib and dexamethasone.

## Conclusion and discussion

The presented case proves the complexity of patients affected by MM, also in the light of possible infectious complications in the SARS-CoV-2 pandemic era. Since our patient contracted the infection in 2020 the vaccination against SARS CoV-2 was not yet available which put her at even greater risk of aggravation. Nevertheless, it demonstrates that adequate treatment with the collaboration of different specialists can lead to a positive outcome in those complicated cases. We believe that further progress in MM therapy as well as SARS-CoV-2 treatment and prevention will improve the prognosis of the patients with MM and severe impairment of renal function who tend to have a greater infection risk and higher mortality rate.

Renal failure is one of the most common presentations of MM. Of note, severe renal impairment in patients with MM is an important factor associated with early mortality [3]. Although the use of novel anti-myeloma agents improved the survival of MM patients with kidney dysfunction yet remain at higher risk for early death [4, 5]. Another critical factor for premature mortality are infections as patients with MM are particularly susceptible to viral and bacterial diseases with a sevenfold higher risk of developing any infection [3, 6]. Considering that patients with decreased renal function are also predisposed to an increased infectious risk, the ones with MM and associated renal impairment present an even greater susceptibility to infections and its complications [7–11].

Since the beginning of the SARS-CoV-2 pandemic several studies reported data suggesting that patients affected by cancer appear to be at increased risk for severe form of COVID-19 and its associated complications, including higher death rate [12–14]. Likewise, available evidence indicates increased risk of severe disease and higher mortality rate in MM patients with SARS-CoV-2 infection [15–18]. The high death ratecan be also explained by the need for discontinuation of haematological therapy: nonetheless it is important not to deviate for an extended period from the normal schedule of treatment to achieve a stable MM remission [19].

In case of MM associated with AKI the current guidelines recommend to start anti-myeloma therapy immediately after confirmation of diagnosis in order to restore renal function [20]. In particular bortezomib-based regimens are recommended (grade A recommendation) [21] as several studies demonstrate high renal recovery and dialysis independence rates as a response to rapid reduction of tumour load by bortezomib [22–24]. Current guidelines of the European Myeloma Network recommend delaying anti-myeloma treatment in case of symptomatic SARS-CoV-2 infection, but at the time of SARS-CoV-2 disease in our patient no guideline was available [25]. Antimyeloma therapy was suspended during the symptomatic infection onset until stabilization of the respiratory status and resumed while the patient was still positive after careful evaluation of risk/benefit ratio with haematology specialist. Such approach was adopted in order to ameliorate the prognosis of the patient by reducing the tumour load and therefore achieving recovery of kidney function, also in consideration of stable improvement of COVID-19 symptoms at the time. As to steroid therapy, it was detrimental and favoured the SARS -CoV-2 onset, but its fulminant clinical course and the recent biopsy proven diagnosis of CN did not allow a significant steroid tapering. Nonetheless, when the patient needed an intensive ventilatory support, steroid therapy allows to counteract the cytokine storm of the acute SARS -CoV-2 pneumonia [26].

Some authors support the use of high cut-off hemodialysis (HCO-HD) or plasmapheresis (PE) in combination with chemotherapy for patients with MM with AKI, but the available evidence is conflicting [22, 27–29]. Leung et al. firstly assessed that those patients who experienced renal function recovery obtained a sFLC reduction > 50% combining chemotherapy (steroids) and PE [30]. Gupta et al. carried out a comprehensive analysis of 3 randomized controlled trials (RCTs) plus 3 retrospective studies (total = 257 pts) where PE was used in the management of AKI during MM. Two out of three RCTs did not show significant differences in survival of the group of patients treated with PE plus chemotherapy compared to the control group (where only chemotherapy was administered). Two out of three RCTs showed a greater percentage of patients who were able to suspend dialysis in the 1^st^ group compared to the 2^nd^, but this result was not reproduced in the 3rd trial which had a higher number of patients [31]. All studies showed a greater recovery of eGFR in patients also treated with PE compared to controls, but only in 2 RCTs and in 1 retrospective study this difference was statistically significant. Nonetheless 3 main concerns affect the use of PE: i) In patients with AKI, dialysis treatment should be associated; ii) PE carries out exchanges on volumes that are not proportionate to the distribution of light chains; iii) Large quantities of plasma and/or albumin are required [31].

As to HCO-HD, one of the concerns regards the risk of albumin loss, especially in case of an intensive HCO-HD regime (such as Birmingham protocol of 8 h daily for 5 days followed by alternate day dialysis through day 21) [32]. Serum albumin within the normal limits is associated with improved survival in patients affected by MM [33]. We explored in depth the efficacy of PEPA in removal of sFLC without albumin loss as possible alternative to HCO-HD. Hutchinson and coll. have already analysed the PEPA adsorbing properties in vitro: it determined a sFLC RR of 55% [34]. Before us a single case report proved the efficacy of PEPA dialyzer in light chain removal: Machiguchi et al. used a previous PEPA blend to remove free lambda light chains in a patient with primary amyloidosis and AKI: a single PEPA filter was used both in HD and HDF during a 37 months follow up, it was efficient in lambda light chain reduction rate but the light chains values on starting dialysis were below 500 mg/L [35]. Therefore, we proposed 2 PEPA dialyzers in series according to Fabbrini et al.: at the 2^nd^ dialysis hour the replacement of the previous exhausted dialyzer takes place [36].

In consideration of our previous positive experience in a single case report and in a cohort of 16 patients, in our patient 2 PEPA dialyzers were used in series during each HDF session with positive outcome and without albumin loss [37, 38].

## Data Availability

The data generated and analyzed in this case are presented within the manuscript.

## Abbreviations

ABG: Arterial blood gas analysis
AKI: Acute Kidney Injury
MM: Multiple Myeloma
CN: Cast nephropathy
eGFR: Estimated glomerular filtration rate
HD: Hemodialysis
HDF: Hemodiafiltration
SARS-CoV-2: Coronavirus Disease 19
sFLC: Serum Free Light Chains
PEPA: Poly Ester Polymer Alloy
HRCT: High Resolution Computed Tomography
PET: Positron Emission Tomography
