# Correction: Acute myeloma kidney and SARS-COV2 infection with dialysis need: never say never - a case report

**DOI:** 10.1186/s12882-023-03301-3

**Published:** 2023-09-08

**Authors:** Gabriele Donati, Agnieszka Przygocka, Fulvia Zappulo, Gisella Vischini, Sabrina Valente, Gaetano La Manna

**Affiliations:** 1 Nephrology Dialysis and Renal Transplantation Unit, Azienda Ospedaliero-Universitaria di Modena. Surgical, Medical, Dental and Morphological Sciences Department (CHIMOMO), University of Modena and Reggio Emilia, Modena, Italy; 2 Nephrology Dialysis and Renal Transplantation Unit, Department of Medical and Surgical Sciences (DIMEC), University of Bologna, IRCCS Azienda Ospedaliero-Universitaria di Bologna, Bologna, Italy; 3 Clinical Pathology, Department of Medical and Surgical Sciences (DIMEC), University of Bologna, Bologna, Italy

**Correction: BMC Nephrology (2023) 24:204**

10.1186/s12882-023-03237-8

Following publication of the original article [1], the authors corrected the 2# and 3# affiliation.

The incorrect affiliations are:

^2^Nephrology Dialysis and Renal Transplantation Unit, Department of Experimental Diagnostic and Specialty Medicine (DIMES), University of Bologna, IRCCS Azienda Ospedaliero Universitaria di Bologna, Via Massarenti 9, 40138 Bologna, Italy

^3^Clinical Pathology, Department of Experimental, Diagnostic and Speciality Medicine (DIMES), University of Bologna, Bologna, Italy

The correct affiliations are:

^2^Nephrology Dialysis and Renal Transplantation Unit, Department of Medical and Surgical Sciences (DIMEC), University of Bologna, IRCCS Azienda Ospedaliero-Universitaria di Bologna, Italy

^3^Clinical Pathology, Department of Medical and Surgical Sciences (DIMEC), University of Bologna, Bologna, Italy
